# Comparison of PAP smear and liquid based cytology as a screening method for cervical carcinoma

**DOI:** 10.12669/pjms.38.7.5742

**Published:** 2022

**Authors:** Mehnaz Khakwani, Rashida Parveen, Maryam Azhar

**Affiliations:** 1 Mehnaz Khakwani, FCPS. Department of Obstetrics and Gyne, Unit-1, Nishtar Medical University, Multan, Pakistan; 2 Rashida Parveen, FCPS. Department of Obstetrics and Gyne, Unit-1, Nishtar Medical University, Multan, Pakistan; 3 Maryam Azhar, MBBS. Department of Obstetrics and Gyne, Unit-1, Nishtar Medical University, Multan, Pakistan

**Keywords:** Cervical carcinoma, Conventional PAP smear, Liquid based cytology

## Abstract

**Objectives:**

To compare conventional PAP smear (CPS) and liquid-based cytology (LBC) for cervical carcinoma screening at a tertiary care hospital of South Punjab, Pakistan.

**Methods:**

This cross-sectional study was conducted at the Department of Obstetrics and Gynecology, Nishtar Hospital, Multan, Pakistan from January 2021 to June 2021. We included a total of 265 women aged between 20 to 65 years who, presented with complaints related to cervical lesion and unhealthy cervix. The CPS and LBC methods were applied for screening of cervical carcinoma. Findings of both CPS and LBC were compared with histopathological findings to find out sensitivity, specificity, positive predictive value and negative predictive value for both techniques.

**Results:**

In a total of 265 women, mean age was noted to be 45.4±6.8 years. White discharge per vagina was the commonest presenting complaint noted in 12 (46.8%) patients. Satisfactory smears were found in significantly more cases with LBC in comparison to CPS (p<0.001). Sensitivity CPS and LBC for the detection of low-grade squamous intraepithelial lesion (LSIL) were found to be 71.8% and 87.2% while for high-grade squamous intraepithelial lesion (HSIL), sensitivity of CPS and LBC were 61.9% and 76.2% respectively. Specificity of CPS and LBC for the detection of LSIL was found to be 97.9% and 98.7% while for HSIL, specificity of CPS and LBC was 98.7% and 99.2% respectively.

**Conclusion:**

In comparison to conventional CPS, LBC was found to be better in terms of adequacy of smear and identification of LSIL and HSIL.

## INTRODUCTION

Cervical cancer is one of the most common types of cancer in women. Recent studies have estimated around five hundred thousand new cases of cervical carcinoma every year while around 80% of these cases are from developing countries.^1^,^2^ Annually, more than 265,000 deaths are linked with cervical carcinoma. In developed countries, morbidity and mortality associated with cervical cancer has declined significantly mainly because of cervical screening. On the other hand, lack of screening in the developing world has resulted in surge in cervical cancer cases in the recent decades.^3^

Prevention and detection of cervical cancer is possible with the help of conventional screening methods like regular pap smear, which is known to be the most commonly adopted method.^4^ Screening of cervical cancer is dependent upon the identification of the squamous intraepithelial lesions that precede the invasive form of cervical cancer. In the past, systemic reports have pointed out towards variation in false negative reports ranging between 6-50% while overall sensitivity patterns are also not very high (between 20 to 50%) with the conventional screening methods.^5^ Due to known limitations of the “conventional Pap smear (CPS)”, “liquid-based cytology (LBC)” was 1^st^ came on view in the 1990s as an alternative approach for the screening of cervical samples.^6^ The LBC uses an improved sampling approach where a collection device is employed for obtaining a large sample that is transferred to a preservative fluid prior to its prepaparation.^7^

Researchers have reported that with the use of LBC, proportion of unsatisfactory samples and time required for the screening have decreased significantly whereas LBC has been found to have higher rates of “high-grade squamous intraepithelial lesion (HSIL)” and glandular lesion, resulting in 14.3% higher diagnosis rates.^8^ A recent study from India has reported sensitivity of CPS to be 63.6% in identification of HSIL in comparison to 72.7% with LBC.^9^ Lack of evidence is found from Pakistan comparing LBC and CPS for the screening of cervical cancer so this study was aimed to compare these two techniques used for cervical carcinoma screening.

## METHODS

The Department of Obstetrics and Gynecology, Nishtar Hospital, Multan, Pakistan from January 2021 to June 2021 was the venue for this cross-sectional study. Approval from “Institutional Ethical Review Board” was acquired (No.23483/NMU&H, dated: 03-12-2020). Informed and written consent was sought from all study participants. The sample size was calculated to be 265 women by using WHO sample size calculator considering sensitivity of CPS for the successful detection of HSIL as 63.6% with confidence level of 95% and margin of error as 6.2%.

A total of 265 women aged between 20 to 65 years, presented with complaints related to cervical lesion and unhealthy cervix were enrolled. All women having pregnancy, postpartum, history of hysterectomy, history of diagnosis or treatment of carcinoma were not enrolled. All women who did not give consent for inclusion in this study were also excluded.

A special proforma was designed to collect all study data. Age, menstrual status and any contraception methods adopted were noted. The pap smears were acquired via Cusco’s speculum examination and detection of any gross abnormalities were recorded. Then, the ayers spatula was utilized for the sampling of the cervix. The material on the Spatula was smeared on two glass slides and immediately fixed in 95% ethyl alcohol for staining with modified Papanicolaou stain and for preparation of conventional smears for microscopic examination. For LBC, cervical brush known as broom was employed for the collection of the samples from the transformation zone of the cervix. The brush head was detached and dropped in the vial containing a specific preservative. Vials containing the specimens were shaken vigorously, and sent to institutional laboratory for CPS and LBC analysis. Findings of both CPS and LBC were compared with histopathological findings.

Data was analyzed by using SPSS version 26.0. Mean and standard deviation (SD) were calculated for quantitative variables while qualitative data was represented as frequencies and percentages. Effect modifiers were stratified and post-stratification chi square test was applied taking p-value ≤0.05 as significant. Sensitivity, specificity, positive predictive value (PPV) and negative predictive value (NPV) for both CPS and LBC were also calculated for the detection of squamous intraepithelial lesions.

## RESULTS

In a total of 265 women, mean age was 45.4±6.8 years. There were 152 (57.4%) women who belonged to rural areas of residence. Socioeconomic status of 152 (57.4%) women was low. White discharge per vaginum (PV) was the commonest presenting complaint followed by lower abdominal pain noted in 124 (46.8%) and 73 (27.5%) patients respectively. Table-I is showing socio-demographic and clinical characteristics of women studied.

**Table I:** Socio-demographic and Clinical Characteristics of Women (n=265).

Characteristics		Number (%) / Mean±SD
Age in years		45.4±6.8 years
Area of Residence	Urban	113 (42.6%)
	Rural	152 (57.4%)
Socioeconomic Status	Lower	152 (57.4%)
	Middle	97 (36.6%)
	Upper	16 (6.0%)
Using Contraception	Barrier	11 (4.0%)
	Oral Contraceptive Pill	8 (2.9%)
	Tubectomy	154 (55.4%)
	None	105 (37.8%)
Clinical Presentation	White Discharge PV	124 (46.8%)
	Lower Abdominal Pain	73 (27.5%)
	Burning Micturition	19 (7.2%)
	Post-Coital Bleeding PV	16 (6.0%)
	Intermenstrual Bleeding PV	33 (12.5%)

Comparison of satisfactory smear collection and endocervical cells detection rates among both methods have showed that LBC had significantly better rates in comparison to LBC (p<0.001).Table-II

**Table II:** Comparison of Satisfactory Smear and Endocervical Cells Detection Rates Between CPS and LBC.

Study Variable	CPS (n = 265)	LBC (n=265)	P-Value
Satisfactory Smear	217 (81.9%)	248 (93.6%)	<0.001
Endorcervical Cells Present	102 (38.5%)	39 (14.7%)	<0.001

“CPS: Conventional Pap smear, LBC: Liquid based cytology”.

Microscopic features of CPS and LBC showed no statistically significant difference and the details are shown in Table-III. Epithelial abnormalities were found among 69 women with CPS and 83 women with LBC.

**Table III:** Microscopic Features between CPS and LBC.

Microscopic Features	CPS (n = 217)	LBC (n=248)	P-Value
Negative for Intraepithelial Lesion or Malignancy	130 (59.9%)	139 (56.0%)	0.6112
Epithelial Abnormalities	69 (31.8%)	83 (33.5%)	
ASCUS	22	27	
LSIL	28	34	
HSIL	13	16	
SCG	4	4	
AGC-NOS	2	2	
Normal	18 (8.3%)	26 (10.5%)	

“CPS: Conventional Pap smear, LBC: Liquid based cytology”

Histopathological findings revealed “cervical intraepithelial neoplasia” (CIN) I, CIN II, CIN III, squamous cell carcinoma (SCC) and chronic cervicitis to be present in 39 (13.6%), 8 (3.0%), 13 (4.9%), 7 (2.6%) and 6 (2.3%) cases respectively.

Sensitivity CPS and LBC for the detection of LSIL were found to be 71.8% and 87.2% while for HSIL, sensitivity of CPS and LBC were 61.9% and 76.2% respectively. Specificity of CPS and LBC for the detection of LSIL were found to be 97.9% and 98.7% while for HSIL, specificity of CPS and LBC were 98.7% and 99.2% respectively. Table-IV is showing sensitivity, specificity, PPV and NPV of CPS and LBC regarding LSIL and HSIL.

**Table IV:** Diagnostic Parameters of CPS and LBC in Detecting Low-Grade Squamous Intraepithelial Lesion and High-Grade Squamous Intraepithelial Lesion.

	LSIL		HSIL	

Parameters	CPS (%)	LBC (%)	CPS (%)	LBC (%)
Sensitivity (%)	71.8	87.2	61.9	76.2
Specificity (%)	97.9	98.7	98.7	99.2
PPV (%)	84.8	91.9	81.2	88.9
NPV (%)	95.5	97.9	96.7	97.9

“LSIL: Low-Grade Squamous Intraepithelial Lesion; HSIL: High-Grade Squamous Intraepithelial Lesion; CPS: Conventional Pap smear, LBC: Liquid based cytology”.

## DISCUSSION

In the past, CPS has been considered as the most adopted method for the screening of cervical cancers but it has always to been regarded as the method that has its own limitations.^10^ False negative reporting with CPS can be attributed to improper sampling, insufficient transferring on glass slide while around 20% of the collected cells are actually smeared on the glass slide.^11^ To address these limitations, an improved slide preparation approach known as LBC was adopted by Maksem et al.^12^ LBC enables uniform cells suspension in a monolayer with the help of centrifuge technique resulting in improvement of specimen adequacy and identification of precursor lesions.^13^

In the present study, we noted that satisfactory smears were found in significantly more samples with LBC in comparison to CPS (81.9% vs. 93.6%, p<0.001). These findings are pretty consistent with what was found by Singh et al where the researchers noted satisfactory smears with CPS and LBC to be 78.8% and 92.5% respectively (p=0.02).^9^ Other researchers have also pointed out significantly better number of satisfactory smears with LBC in comparison to CPS.^14^ Drying artefact and cytolysis are very less with the use of LBC as immersion of cells in liquid fixative and specimen is significantly better because of absence of influencing factors like blood, mucus and inflammatory cells.^15^ Higher number of unsatisfactory smears with CPS could be because of thick smear which is not the issue in LBC because of even distribution of cells.

We noted endorcervical cells detection in CPS and LBC methods to be in 38.5% and 14.7% cases respectively (p<0.001). These findings are consistent to what has been reported in the literature which could be because of the reasons that smear are collected by split sample methods where slide for CPS are prepared first and then same brush head is suspended in the preservative fluid after detachment in LBC method.^16^ Endocervical cells are less likely to be shifted to the vial as those are more likely to have been trapped in the endocervical mucus which is held by the collection hairs of the cervix brush and so can disperse in the fixative. Detection of epithelial cell abnormalities wit CPS and LBC were not found to be significantly different (p=0.6112). Similar results have been reported by other researchers before.^15^,^17^

In the present research, sensitivity of LBC for the detection of histopathologically proven LSIL/CIN-I was much higher (87.2%) in comparison to CPS (71.8%). A study by Singh et al found sensitivity of CPS and LBC for the identification of LSIL to be 71.4% and 78.4% respectively.^9^ Langatto et al noted sensitivity of CPS and LBC for the identification of LSIL to be 49.8% and 70.0% respectively.^18^ Beerman et al noted relatively high and similar sensitivities of CPS and LBC for the identification of LSIL as 92.0% and 96.2% respectively.^19^ In the present research, sensitivity of LBC for the detection of HSIL/CIN II and CIN III was much higher (76.2%) in comparison to CPS (61.9%). Singh et al found sensitivity of CPS and LBC for the identification of HSIL to be 63.6% and 72.7% respectively.^9^ Langatto et al noted sensitivity of CPS and LBC for the identification of HSIL to be 728% and 91.3% respectively.^18^ Bergeron et al noted relatively similar sensitivities of CPS and LBC for the identification of HSIL as 82% and 86% respectively.^20^ Specificity patterns of both CPS and LBC were quite high and similar regarding identification of LSIL and HSIL. A recent study by Singh et al found sensitivity and specificity of LBC to be significantly more than CPS for the evaluation of cervical cytology.^21^

### Limitations

As total number of cases were relatively small so our results cannot be generalized and might differ if a similar study is conducted on a large sample base. We were unable to do HPV testing to find out the prevalence of HPV in the patients who were found to have squamous and glandular lesions.

## CONCLUSION

In comparison to conventional CPS, LBC was found to be better in terms of adequacy of smear and identification of LSIL and HSIL. LBC can be used instead of conventional cytology methods for the screening of cervical carcinoma.

### Authors’ Contribution:

**MK:** Conceived, Responsible for data’s integrity and authenticity.

**RP:** Data Collection, Drafting.

**MA:** Data Collection, Literature Review.
